# A hybrid algorithm for coupling partial differential equation and compartment-based dynamics

**DOI:** 10.1098/rsif.2016.0335

**Published:** 2016-09

**Authors:** Jonathan U. Harrison, Christian A. Yates

**Affiliations:** 1 Wolfson Centre for Mathematical Biology, Mathematical Institute, University of Oxford, Andrew Wiles Building, Radcliffe Observatory Quarter, Woodstock Road, Oxford OX2 6GG, UK; 2 Department of Mathematical Sciences, University of Bath, Claverton Down, Bath BA2 7AY, UK

**Keywords:** multiscale, hybrid algorithms, reaction–diffusion, stochastic, deterministic

## Abstract

Stochastic simulation methods can be applied successfully to model exact spatio-temporally resolved reaction–diffusion systems. However, in many cases, these methods can quickly become extremely computationally intensive with increasing particle numbers. An alternative description of many of these systems can be derived in the diffusive limit as a deterministic, continuum system of partial differential equations (PDEs). Although the numerical solution of such PDEs is, in general, much more efficient than the full stochastic simulation, the deterministic continuum description is generally not valid when copy numbers are low and stochastic effects dominate. Therefore, to take advantage of the benefits of both of these types of models, each of which may be appropriate in different parts of a spatial domain, we have developed an algorithm that can be used to couple these two types of model together. This hybrid coupling algorithm uses an overlap region between the two modelling regimes. By coupling fluxes at one end of the interface and using a concentration-matching condition at the other end, we ensure that mass is appropriately transferred between PDE- and compartment-based regimes. Our methodology gives notable reductions in simulation time in comparison with using a fully stochastic model, while maintaining the important stochastic features of the system and providing detail in appropriate areas of the domain. We test our hybrid methodology robustly by applying it to several biologically motivated problems including diffusion and morphogen gradient formation. Our analysis shows that the resulting error is small, unbiased and does not grow over time.

## 1. Introduction

Multiscale modelling challenges occur frequently throughout cellular biology and in the context of cell migration. Spatial reaction–diffusion models can be used to describe, either deterministically or stochastically, various biological phenomena. These include actin dynamics in filopodia [1], calcium signalling [2] and chemisorption of polymers [3]. In many cases, it may be beneficial to use a multiscale approach to modelling using different descriptions in different spatial regions. In this article, we will set out a method for coupling together a continuum deterministic description and a discrete stochastic description.

Commonly, continuum approaches using partial differential equations (PDEs) are adopted to model biological systems [4]. These equations can either be solved analytically (in some cases) or simulated numerically. Results using this methodology are relatively fast to calculate computationally. However, for systems with small numbers of molecules the results obtained using deterministic methods may not always capture the behaviour of a stochastic system appropriately, especially in situations where molecular numbers are low and interactions are nonlinear. For example, in a system with multiple steady states such as the canonical model of Schlögl [5], a deterministic model fails to capture the switching behaviour between the steady states seen in a stochastic model. In general, PDE models break down when the number of molecules present is very low and stochastic effects dominate [6]. Although deterministic models may provide useful information about average behaviour (in the case of linear systems), they cannot offer a full description of every system. Thus, in the case where copy numbers are low, the best description will be afforded by a stochastic model. There are two main types of stochastic models used for reaction–diffusion equations [7]: off-lattice methods and on-lattice compartment-based methods. We will focus on compartment-based methods, which generally offer a coarser description than their off-lattice counterparts.

When simulating a system using a compartment-based stochastic model (also known as a position-jump model), the computational cost of the simulations can become prohibitive if the number of particles in the system is high. A computationally efficient continuum model may be more appropriate in this scenario. Thus, in situations where particle concentrations vary widely across the domain there may be advantages to using a continuum PDE model in the region of the spatial domain where particle numbers are high and a discrete stochastic model elsewhere. Moreover, detail is often only required in a certain part of the domain and thus a spatial-hybrid model may be most appropriate [1,3,7–10]. Such a hybrid model would allow an accurate representation of the reaction–diffusion dynamics in the region where this is required but minimizes the computational resources needed to perform the calculation by using less detailed, more efficient methods in regions of the domain where detail is not required.

Previously, Flekkøy *et al*. [8] have developed a hybrid model that links a PDE-based model to the motion of random walkers on a lattice. Motivated by heat transport around a facture in a solid, Flekkøy *et al*. [8] choose a detailed description of the particle dynamics coupled to a coarse-grained PDE model: the lattice spacing used to solve the PDE is larger than in the particle-based region. More recently, PDE-to-compartment hybrid methods have been developed which employ a region of the PDE regime in which particles are represented using both the compartment- and PDE-based modelling regimes simultaneously [11,12]. The duality of these so-called ‘pseudo-compartment methods' allows for particles to behave correctly as they cross individually between the two different regimes because particles can jump into their neighbouring compartment according to standard compartment-based rules for diffusion.

Our hybrid modelling regime employs a PDE mesh that is significantly (and arbitrarily) finer than the lattice in the compartment-based region. This choice is natural in many situations, including in a biological context, where we are choosing to use the PDE model in regions of high population to offer improved computational efficiency. Taking a fine mesh will not prove computationally prohibitive compared with the stochastic model, but allows us to make the numerical solution of the PDE arbitrarily accurate. Methodologies with coarser or equal PDE spacing relative to compartment spacing [8,12] are open to questions about what exactly the ‘PDE regime’ represents given its resolution and accuracy are restricted by the resolution of the compartment-based method.

Our approach to coupling of deterministic PDE-based and stochastic compartment-based regions employs an overlap region where both modelling descriptions are valid. This overlap region can contain multiple compartments if desired. The method that we have developed relies upon specifying a Dirichlet-type condition between the two models at one interface at the edge of the PDE-based region and dictating the correct flux of PDE on compartments at the other interface. This fixes the boundary conditions at the interfaces between each of the regions.

In the remainder of this article, we describe and explore our novel hybrid coupling algorithm in detail and illustrate the effectiveness of the method. In §2.2, we present the hybrid method in full and justify the coupling conditions chosen. Thereafter, in §3.1, we demonstrate the appropriate behaviour of our method through its application to systems of diffusing particles with various extreme initial conditions (chosen specifically to test the algorithm) and a biologically motivated example: the formation of a morphogen gradient. We apply the model to a travelling wave example in §3.1.3 and introduce an adaptive interface between the modelling regimes in §3.1.4. We present detailed simulation-time comparisons of the hybrid model with the fully stochastic model for our test problems at the end of §3.1.4, which explicitly demonstrate the improved efficiency of our hybrid method. The fidelity of the algorithm's performance is then examined and the error (with respect to a range of model parameters) analysed in §3.2. We verify in §3.3 that the hybrid coupling algorithm gives results which match the variances across the interface, as well as the mean behaviour. We conclude in §4, with a discussion of the potential advantages of this hybrid method in relation to other existing methods.

## 2. Methods

### 2.1. The domain

**Figure 1.**
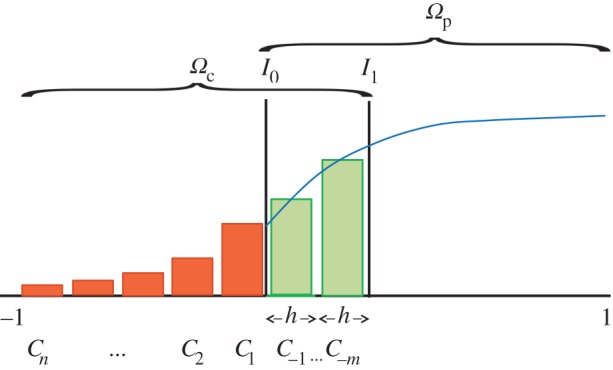
The domain *Ω* showing the division into a compartment-based region *Ω*_c_ on [−1, *I*_1_] and a PDE-based region *Ω*_p_ on [*I*_0_, 1] with an overlap region where both model descriptions are valid on [*I*_0_, *I*_1_]. Orange bars represent the number of particles in the fully compartment-based regime, green bars represent the number of particles in each compartment of the overlap region, and the blue curve represents the solution of the PDE.

Suppose, arbitrarily, we have a domain *Ω* = [−1, 1] which we divide into a region *Ω*_c_ in which we use a compartment-based, stochastic model and a region *Ω*_p_ in which we use a deterministic, PDE-based model. A characterizing feature of our hybrid methodology is an overlap region (shown in figure 1) in which both modelling regimes are simultaneously valid descriptions (i.e. 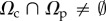). Either side of the overlap region, we have interfaces *I*_0_ and *I*_1_ (figure 1). In a similar context, it has been demonstrated that an overlap region is required to give the appropriate variance for a model coupling a Brownian motion particle-based description and a PDE-based model [3].

In *Ω*_c_, the domain is split into compartments of width *h*, where the *k*th compartment occupies the region 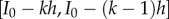 for 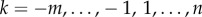. The *m* compartments labelled –*m*, … ,−1 are situated in the overlap region and the *n* compartments (note there is no compartment 0) labelled 1, … , *n* are in [−1, *I*_0_]. The labelling of compartments is illustrated in figure 1. We assume particles are well mixed within these compartments.

A continuum description of diffusion, as assumed when modelling with a PDE, requires sufficiently high particle numbers. For low concentrations, this assumption breaks down. If the concentration of particles is *u*(*x*, *t*), given a total of *N* particles in the system, then we can relate the probability of finding any particular particle in the system, *p*_p_(*x*, *t*), to the concentration as 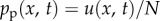. This probability density remains well defined even at low particle copy numbers, when we cannot interpret the PDE as a concentration but are able to view it as a probability. Therefore, the probability to find each of *N* particles in the PDE region in a given interval [*x*, *x* + d*x*] at a certain time *t* is 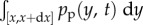. The expected number of particles in a subset, *ω*, of the PDE domain, *Ω*_p_, is given by 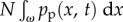. We will use *u_k_*(*t*) where *k* = 1, … , *K* + 1 to denote the PDE density at the *k*th PDE lattice point in the finite difference discretization of the PDE required for our hybrid algorithm.

For the compartment-based regime, let *p*_c_(*x*, *t*) (defined initially only at the centre of compartments) be the probability of finding one of the identically initialized particles at position *x* at time *t*. Because each compartment is well mixed, we can describe the evolution of *p*_c_(*x*, *t*) using the reaction–diffusion master equation [13]. We will also use the notation 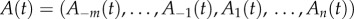 to represent the distribution of particle numbers across compartments.

### 2.2. The coupling algorithm

We now describe an algorithm, which couples the two regimes together. Informally, the coupling is achieved by setting the value of the PDE lattice point at *I*_0_ to the average of the adjacent compartment populations in *Ω*_c_ and using the gradient in the PDE-based region *Ω*_p_ to give a rate of jumping across the interface *I*_1_ for the compartment-based regime.

In what follows, we specify and justify these coupling conditions mathematically. These conditions are analogous to a Neumann condition for the compartments at *I*_1_ and a Dirichlet condition for the PDE at *I*_0_. We aim to apply an appropriate flux of particles to and from *Ω*_c_ based on the PDE profile across the interface *I*_1_, which will ensure that the gradients of the different modelling regimes agree. Feasibly, if this were the only condition, situations could arise where the gradients of the two regimes agree, but there is a notable discontinuity in the values of the density between descriptions. To prevent this, we enforce a boundary condition on the PDE requiring the density on the lattice point at *I*_0_ to match an average of the density of the surrounding compartments. These conditions are chosen to maintain both the continuity of flux and mass/density across the discrete–continuum interface. A similar approach has been employed for a hybrid fluctuating hydrodynamics model [14].

In order to justify our coupling, first consider the Dirichlet matching condition at *I*_0_, where we specify the PDE density in terms of particle numbers2.1
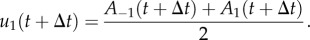
Writing this in terms of the analogous probability densities, we have2.2
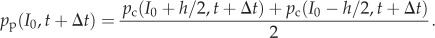
Extending *p*_c_ to continuous space (an approach used similarly in previous work [7,15,16]) and Taylor expanding the terms on the right-hand side (RHS) of equation (2.2) to first order, we find that
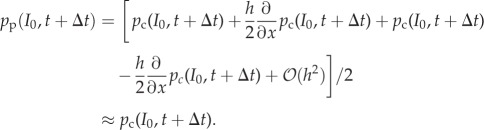
This suggests that matching condition (2.1) ensures agreement between the solution of the PDE and compartment-based particle numbers at *I*_0_. The agreement will become exact in the limit *h* → 0.

For the condition at *I*_1_, we want to match the flux across *I*_1_ in the compartment-based regime to that in the PDE regime. We will show that by enforcing the matched flux condition, the probability density for the compartment-based region evolves according to the diffusion equation in the limit of small compartment size.

We begin by writing down the master equation [13] for the probability density of a single particle at compartment −*m*, adjacent to the interface, *I*_1_:2.3
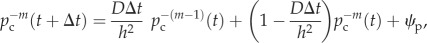
where 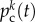 is shorthand for 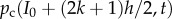 and describes the probability density for a single diffusing particle to be found in the *k*th compartment at time *t*. Here, *ψ*_p_ is the flux imposed (as part of the hybrid algorithm) on compartment −*m* from the right. If there were compartments to the right of the compartment labelled −*m* (i.e. –(*m* + 1), etc. (figure 1)) the true net flux would simply be2.4
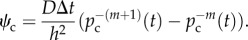
Instead, we must approximate the true flux, *ψ*_c_, by an ansatz derived from the PDE, *ψ*_p_ as follows.

Suppose that the *l*th lattice point of the PDE lies on the interface *I*_1_, and *w* is the ratio of spacing between the compartment size, *h*, in *Ω*_c_ and the PDE finite difference lattice size, 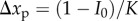, such that 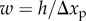.

In order to approximate the flux across the interface with *ψ*_p_, we must approximate the density at the centre of compartments adjacent to the interface *I*_1_, based on the density of the PDE. In general, the PDE mesh will not coincide with the centre of the compartments. Therefore, we apply a linear interpolation of the density based on the PDE mesh points closest to the centres of the relevant compartments. The linear interpolation is chosen because it is the simplest method, but provides sufficient accuracy to approximate the flux across the interface.

We interpolate the density in *Ω*_p_ at the centre of the −*m*th compartment by
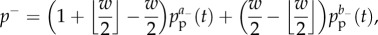
where 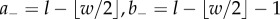. Imagine an extra compartment –(*m* + 1) to the right of *I*_1_. We could interpolate the density at the centre of this ghost compartment using a similar expression
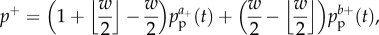
where 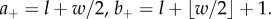


Given these interpolations of the PDE density at the centre of compartments, we can approximate the diffusive flux across the interface and consequently set2.5
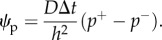
Substituting this into equation (2.3) gives2.6
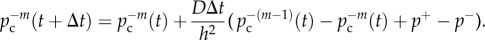
Upon rearrangement this implies
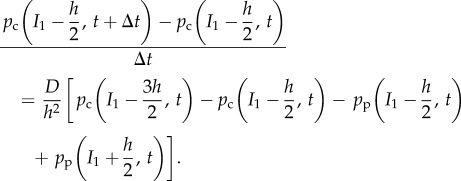
In order to demonstrate the veracity of our choice of *ψ*_p_, we extend *p*_c_ to be a continuous function of space and Taylor expand terms on the RHS in space about the centre of the −*m*th compartment (i.e. *I*_1_ − *h*/2). Taylor expanding 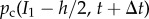 in time and taking the diffusive limit, we find we recapitulate the diffusion equation for the probability density at *I*_1_ − *h*/2 if 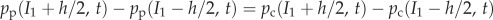 or equivalently *ψ*_p_ = *ψ*_c_. Consequently, this indicates that the flux *ψ*_p_ given by equation (2.5) is an appropriate boundary condition for the compartment-based model.

Algorithm 1. **Time-based hybrid algorithm for stochastic reaction–diffusion simulations using a compartment-based region and an overlapping PDE-based region.**

(i) Initialize number of particles *A_k_*, 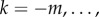 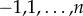 in compartments in *Ω*_c_ and apply consistent initial conditions in *Ω*_p_.
(ii) Select the compartment-based time step Δ*t*, such that the probability of more than one event occurring per time step is *O*(Δ*t*^2^), and a maximum duration of the simulation, *T*_final_. Set *t*:= 0.
(iii) Calculate *ψ* = *Nψ*_p_, where *ψ*_p_ is as in equation (2.5).
  Draw a uniform random number *r*_1_.
  If *r*_1_ < |*ψ*|, then update 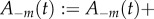 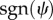.
(iv) Calculate a uniform random number *r*_2_.
  If *r*_2_ < *α*_0_Δ*t*, where *α*_0_ is the total propensity of the ‘reaction’ events in the compartment-based regime, then a reaction occurs in that time step.
(v) If a ‘reaction’ occurs, generate a uniform random number *r*_3_, and find *j* such that 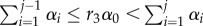.
  Update number of particles in each compartment according to chosen reaction, *j*.
(vi) Update time such that 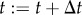.
(vii) Update PDE region *Ω*_p_ using an appropriate numerical method. Apply the boundary condition at the right-hand boundary and the coupling condition at *I*_0_ as follows: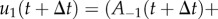 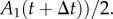
(viii) If *t* < *T*_final_, then go back to step (iii). Else, end.

Given our two matching conditions at either end of the interface, the hybrid algorithm can be implemented in a time-driven sense as given in Algorithm 1. Note the factor of *N* in the calculation of *ψ* at step (iii) is due to the scaling between concentration and the probability distribution for a single particle. Taking sgn(*ψ*) in step (iii) corresponds to either adding a particle into the −*m*th compartment due to flux into *Ω*_c_ when sgn(*ψ*) = + 1 or removing a particle due to flux out of *Ω*_c_ when sgn(*ψ*) = −1.

Both ‘time-based’ and ‘event-based’ versions of the hybrid coupling algorithm are possible [3]. The main difference between these is that the time-based algorithm uses a fixed time step Δ*t* to update both *Ω*_c_ and *Ω*_p_, while the event-based algorithm steps forward to the next reaction in *Ω*_c_ [17], while still fixing a maximum time step in *Ω*_p_ for updating the PDE. For systems with large numbers of particles, the event-based algorithm will be more efficient as it allows the use of larger time steps in the stochastic regime so fewer steps of the algorithm are required. However, for simplicity, we present here the time-based version, Algorithm 1.

## 3. Results

### 3.1. Numerical simulations

#### 3.1.1. Test problem: diffusion

We will begin our examination of practical applications of the hybrid coupling algorithm by applying the method to a test problem in which particles diffuse with diffusion constant *D*. With large copy numbers of particles in the system, the density of diffusing particles, *u*(*x*, *t*), is governed by the diffusion equation3.1
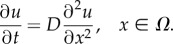
Adding reactions to this system should not affect the boundary behaviour directly and therefore it is sufficient to test our model on a problem of this type [15]. As previously specified (but without loss of generality), our domain is *Ω* = [−1, 1] with zero flux boundary conditions at both ends. This domain is divided into a deterministic PDE-based region and a stochastic compartment-based region as required by the hybrid coupling algorithm. We choose *Ω*_c_ = [−1, 0.1], *Ω*_p_ = [0, 1]. The left-hand interface of the overlap region is at *I*_0_ = 0 while the right-hand interface of the overlap region lies at *I*_1_ = 0.1.

We consider three different initial conditions, *ϕ*(*x*): a uniform initial condition, demonstrating that the algorithm can maintain an equilibrium state, a step function with all the mass in [0, 1], that is3.2
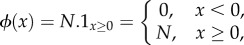
and a step function with all the mass in [−1, 0], that is3.3
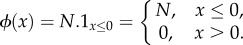
These provide a robust test of our hybrid algorithm in a variety of different scenarios, showing it can maintain net flux from each region to the other. We note that the initial condition in (3.3) is used here to stress test the algorithm under extreme circumstances, and does not correspond to a situation where it would be appropriate to apply this methodology. Generally, the PDE representation should be used to model regions of high density and the compartment-based representation to model regions of lower density.

We have performed simulations of the hybrid model, using the three different initial conditions described above. We also present the analytical solutions of the mean-field diffusion equation. In particular, suppose that all the mass is initially in [0,1], as in (3.2). Using a Green's function and an infinite series of images at the boundaries, we obtain an analytical solution to equation (3.1) of the form3.4
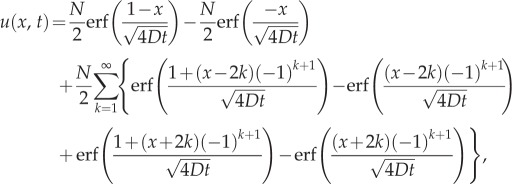
where we have written the solution in terms of error functions. The solution for the initial condition of a step function with all the mass in [−1, 0] (as in equation (3.3)) can be obtained by symmetry from equation (3.4). These solutions are used in figures 3 and 4, respectively.

**Figure 2.**
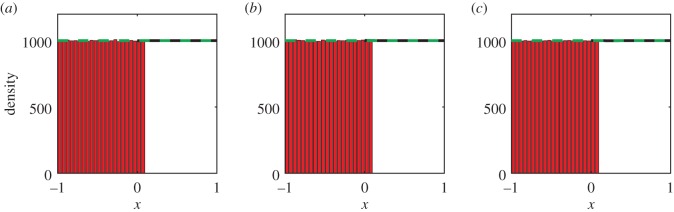
Simulating simple diffusion starting from a uniform distribution of mass throughout the domain *Ω*. Panels (*a*), (*b*) and (*c*) show the particle density at times *t* = 0.1, *t* = 1 and *t* = 10, respectively. Simulations are performed using the hybrid coupling algorithm set out in Algorithm 1. Parameters used are *D* = 0.025, Δ*t* = 0.001, *h* = 0.05, Δ*x*_p_ = 0.01 and the simulation results are averaged over 100 repeats. The black line represents the density in *Ω*_p_ and the red bars represent the particle density in *Ω*_c_. The dashed green line shows the (trivial) analytic solution. (Online version in colour.)

**Figure 3.**
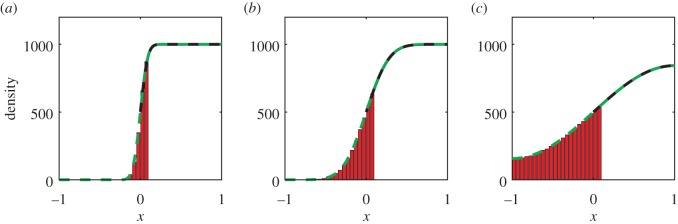
Simulating simple diffusion starting from a step function with mass in [0, 1]. Panels (*a*), (*b*) and (*c*) show the particle density at times *t* = 0.1, *t* = 1 and *t* = 10, respectively. Simulations are performed using the hybrid coupling algorithm set out in Algorithm 1. Parameters, repeats and figure descriptions are as for figure 2. (Online version in colour.)

**Figure 4.**
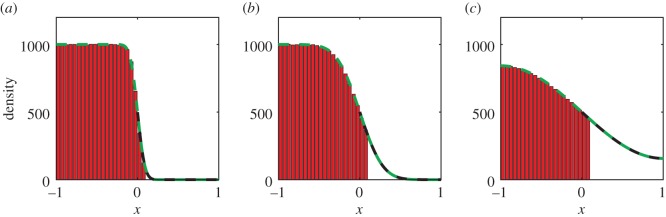
Simulating simple diffusion starting from a step function with mass in [−1, 0]. Panels (*a*), (*b*) and (*c*) show the particle density at times *t* = 0.1, *t* = 1 and *t* = 10, respectively. Simulations are performed using the hybrid coupling algorithm set out in Algorithm 1. Parameters, repeats and figure descriptions are as for figure 2. (Online version in colour.)

Comparisons between our hybrid model and the mean-field analytical solution are shown in figures 2–4 for a range of times. Agreement is observed between the simulated results and the analytic solutions.

**Figure 5.**
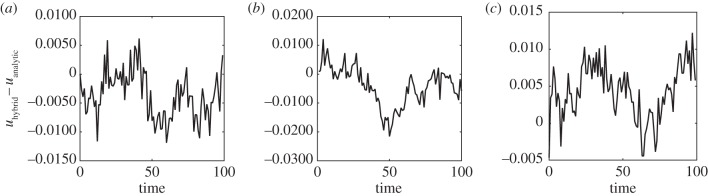
The evolution over time of the error obtained from simulations using the hybrid method with parameters as in figure 4. Panel (*a*) employed a uniform distribution of mass throughout the domain as the initial condition, panel (*b*) a step function with mass in *Ω*_p_, and panel (*c*) a step function with mass in *Ω*_c_. The error is calculated as the difference between the average density given by the hybrid model over 100 repeats and the deterministic expected value of the density.

Quantitative comparisons of the simulations from the hybrid model with the analytic solutions showing the behaviour of the error over time can be seen in figure 5. We compute the error as a sum across the entire spatial domain *Ω* of absolute values of the difference between the average of the hybrid model and the analytic mean-field solutions. This difference is computed at the centre of each region of width *h*, in both *Ω*_c_ and *Ω*_p_. The resulting stochastic error is normalized by the total number of particles in the system. There is no long-term bias in the errors, and crucially, in each case, the magnitude of the absolute error does not increase over time. This demonstrates quantitatively the agreement between the two modelling regimes.

#### 3.1.2. Test problem: morphogen gradient

We also apply our model to another test problem: the formation of a morphogen gradient. For this problem, we use the same domain and partitioning as before. Morphogen molecules are produced at rate *J* at *x* = 1 and throughout the domain morphogen molecules decay with constant rate *μ* and diffuse with diffusion coefficient *D*. When there are sufficiently many molecules in the system, we expect the density of molecules, *u*(*x*, *t*), to be governed by the following PDE:3.5
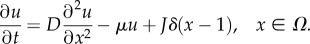
We apply zero flux conditions at the boundaries and initially we assume there are no molecules in the system.

**Figure 6.**
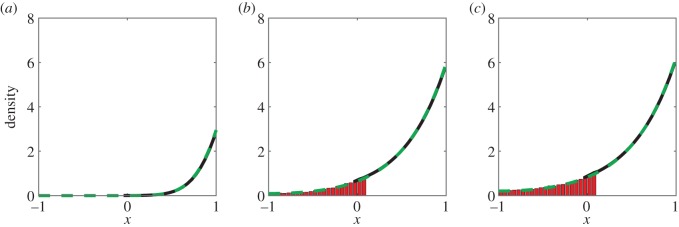
Averaged simulations of the morphogen gradient system with the hybrid algorithm (using equation (3.5) in *Ω*_p_ and stochastic simulations of the reaction scheme in *Ω*_c_) compared to the analytical solution of (3.5). Initially the domain is empty. Simulations are performed up to *t* = 20 and averaged over 100 repeats with parameters *D* = 0.05, *μ* = 0.2, *J* = 125, *h* = 0.05, Δ*x*_p_ = 0.01. Panels (*a*), (*b*) and (*c*) show the particle density at *t* = 1, *t* = 10 and *t* = 20, respectively. Figure descriptions are as for figure 2. (Online version in colour.)

The results of simulating this morphogen system are shown in figure 6. The system was simulated up until *t* = 20 after which point the system had approached steady state. Good agreement can be seen between the hybrid simulation algorithm and the analytical solution of (3.5).

#### 3.1.3. Test problem: travelling wave

The occurrence of travelling waves is common throughout the natural world: they describe a variety of phenomena from propagation of genes in a population [18], to epidemic outbreaks [19], and in the FitzHugh–Nagumo equations for a nerve axon pulse [20].

One commonly used model for a travelling wavefront is the Fisher–KPP equation3.6
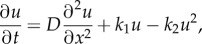
where *D* is the diffusion coefficient, and *k*_1_ and *k*_2_ are reaction rates. This is a nonlinear reaction–diffusion equation for the concentration or population density *u* in one dimension. It can be shown that this results in the formation of a travelling front with a minimum wave speed of 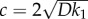, given continuous initial conditions with compact support [21].

Consider the reversible chemical reaction3.7
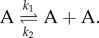
Using the law of mass action in a deterministic setting [21] and including diffusion effects results in the Fisher–KPP equation (3.6) as a description of the evolution of the chemical concentration. To investigate stochastic simulations of the propagation of travelling waves, we can interpret the reaction system (3.7) in a stochastic sense [9]. The stochastic simulations of wavefront propagation do not generally match the deterministic models, with stochastic models resulting in a different wave speed than given by the deterministic model and different speeds given depending on the stochastic model used [22]. The wave speed in the stochastic models approaches that of the deterministic model in the continuum limit of many particles, but does so relatively slowly with 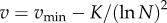, where *v*_min_ is the minimum velocity for the deterministic model, *K* is a constant and *N* is the total number of molecules [23]. By considering moments of the appropriate chemical master equation, we obtain a hierarchy of coupled equations, where the *k*th moment depends upon the (*k* + 1)th moment [24]. In order to obtain a closed system we must make a closure approximation. The degree of agreement between the deterministic and stochastic descriptions will depend on the validity of this closure assumption.

We note that the nature of reaction scheme (3.7) means that population growth in compartments ahead of the wavefront does not begin until there is at least one particle present in that compartment. The discretization of particles in the stochastic model, therefore, restricts the progress of the wave and results in the lower wave speed in comparison to the deterministic interpretation [25].

Given that we do not expect the stochastic model to correspond to the deterministic model in the mean-field we will use a fully stochastic compartment-based description of the system for comparison with our hybrid system in order to determine its accuracy (as opposed to the PDE description which represented the mean-field behaviour of the previous test systems). We expect to make computational savings by using a PDE to describe the mean-field behaviour behind the wave whilst using the stochastic compartment-based model to simulate behaviour at the wavefront and ahead of the wave, which determines the wave speed.

Applications of hybrid models to travelling waves have been made in previous work. Moro [9] has successfully demonstrated such a model, using a flux-based approach similar to that of Flekkøy *et al*. [8]. This hybrid model was then used to confirm the scaling of the velocity correction for the stochastic mesoscopic model. Further to this, an adaptive version of the two-regime method has also been applied to a travelling wave problem [22]. This model couples a microscopic Brownian motion based description to a mesoscopic compartment-based description, as in the original two-regime method [7]. In addition, the interface between the two regions is, in this case, allowed to move adaptively following the propagation of the front [22]. This enables the microscopic description to represent the most appropriate region of the domain, following the front of the wave, with the less computationally intensive mesoscopic description remaining behind the wave.

**Figure 7.**
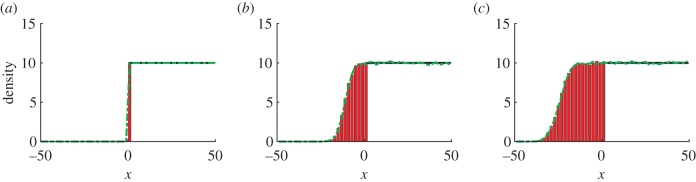
Simulating a travelling wave using the hybrid model, and the fully stochastic scheme (3.7). The results shown have been averaged over 1000 repeats. Parameters used are *D* = 1, *h* = 2, Δ*x*_p_ = 0.5, *k*_1_ = 1, *k*_2_ = 0.1. Panels (*a*), (*b*) and (*c*) show the particle density at times *t* = 0, *t* = 10 and *t* = 20, respectively. The green dashed line shows the result of fully stochastic simulations while the red histogram and black line show the result of the hybrid model in the compartment-based and PDE-based regions, respectively. (Online version in colour.)

We demonstrate that our hybrid model can be applied successfully to a travelling wave using a fixed overlap region between the models, taking the domain as *Ω* = [−*L*, *L*] where *L* = 50, with an overlap region at [0, 2]. Consequently, we have *Ω*_c_ = [−50, 2] while *Ω*_p_ = [0, 50]. We take our initial condition as a step function: 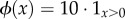. The results of simulations are displayed in figure 7, showing the close agreement between the hybrid model and the fully stochastic model. The hybrid model accurately captures the stochastic behaviour at the front of the wave that is missed by the fully PDE-based model.

An important measure when investigating stochastic simulations of reaction system (3.7) is the resulting wave speed. It can be difficult with a stochastic model to specify exactly where the wavefront is at a given time and to quantify exactly how fast it is moving, because there will inevitably be noise in the results of simulations [24]. We choose to use the method outlined by Robinson *et al*. [22], which considers the rate of change of the total mass, *M*(*t*), in the system. For times *t*_2_ and *t*_1_, we take3.8
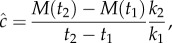
where the factor *k*_2_/*k*_1_ is necessary since the height of the wave will approach *k*_1_/*k*_2_. Dividing the rate of change of mass by this factor gives a measure of how fast the wave is propagating through the domain.

**Figure 8.**
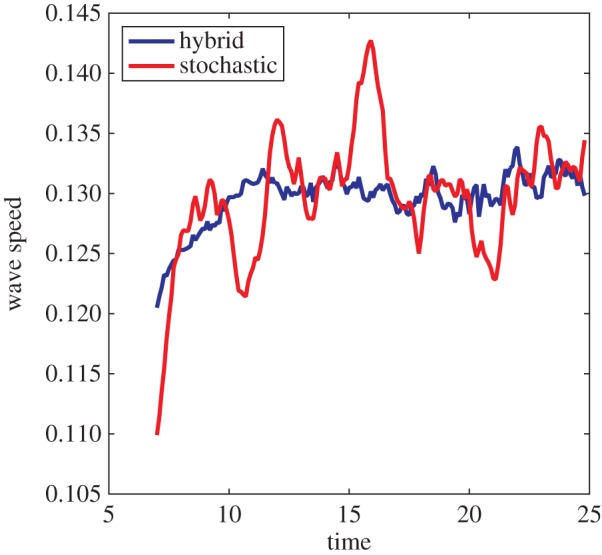
A comparison of the wave speeds resulting from simulations of a travelling wave using a fully stochastic model (shown in red/light grey) and the hybrid model (shown in blue/dark grey). The parameters for the simulations are the same as in figure 7, averaging over 1000 repeats as used previously. The wave speed was estimated using equation (3.8) at regular time intervals and smoothed with a moving average across five time units. The moving average is computed as a mean of the speeds across each five time unit window. (Online version in colour.)

Figure 8 shows a comparison between the wave speeds obtained from the fully stochastic compartment-based model, the hybrid model and the deterministic PDE model. There is more variation in the fully stochastic model since the PDE part of the hybrid model acts to dampen the fluctuations in the stochastic part of the model. Good agreement is seen between the wave speeds of the two models as estimated by a moving average of the wave-speed estimates, after an initial transient. The slower initial wave speed observed in both models is explained by the steep initial condition, which first needs to approach the profile of the travelling wave before it starts to move at constant speed.

#### 3.1.4. Adaptive interface via a local detection criterion

In certain situations, as with the travelling wave presented in the previous section, the region of interest with lower particle numbers changes position dynamically. In order to capture most effectively the detail in this area while reducing the computational requirements it will be useful to have an interface that also changes position, so that regions with higher particle numbers can more often be modelled using the PDE. To ensure that the interface moves correctly, we initiate moves of the interface adaptively based on a detection condition of the particle density near the interface. Moving the interface should also prevent unnecessary simulation of large particle numbers using the stochastic regime in regions where this is not required; for example, behind the wavefront for larger times in the travelling wave model (figure 7*c*).

Such adaptive interfaces have previously been implemented in hybrid models [12] and in several works [22,26] based on the previously mentioned two-regime method [7]. The two-regime method implements a coupling between a compartment-based stochastic model and a molecular based stochastic model. In the adaptive two-regime method [22], the interface between the two models moves adaptively in increments of the compartment width *h*. The moves are made to keep the density of particles below a certain threshold *u*_max_. If the density of the particles in the compartment adjacent to the interface is above *u*_max_, then the interface is moved into the compartment-based region. Conversely, if the density in the molecular region is below another threshold then the interface is moved into the molecular region. This threshold is chosen as *u*_max_ − *δu*, where *δu* is a small (constant) increment, to prevent unnecessary fluctuations in the position of the interface due to the stochasticity of the system [26]. For similar reasons, the condition for updating the position of the interface is not checked every time step but after a fixed number of time steps to prevent errors resulting from moving the interface too frequently [26].

We choose to move the interface only by small increments equal to the compartment width *h* after each successful check of a local detection criterion. This criterion is checked at intervals of *η* steps of the algorithm. The requirement for moving the interface is that the density in both the compartment-based region and PDE-based region near the interface must be either above *u*_max_ or below *u*_max_ − *δu*. Specifically, we check the compartments either side of interface *I*_0_ in *Ω*_c_ and PDE points at equivalent positions either side of the interface *I*_1_.

**Figure 9.**
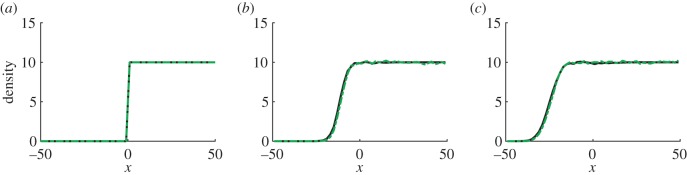
Simulating a travelling wave using the adaptive hybrid algorithm. Parameters used are as described in figure 7, with *u*_max_ = 10.5, *δu* = 1.0, *η* = 50 for adaptive movements of the interface. The black line shows the results of the adaptive hybrid algorithm, while the dashed green line shows the fully stochastic model. Panels (*a*), (*b*) and (*c*) show the particle density at times *t* = 0, *t* = 10 and *t* = 20, respectively. (Online version in colour.)

In the particular case of the travelling wave considered in §3.1.3, it is important that we keep the entire front of the wave in *Ω*_c_, because it is the description governing the wavefront that dictates the wave speed. To ensure this, we take *u*_max_ = 10.5, *δu* = 1.0 for model parameters as in figure 9.

When we have performed several iterations of the hybrid adaptive algorithm and wish to take an average of the results we encounter some difficulties. After a full iteration of the algorithm has been completed, the interface between the models will have, in general, changed position following the wavefront. However, upon repeating the iteration, the position of the interface may have changed by a different amount. This is due to the stochastic nature of the process that we are simulating. We note that in the overlap region both of the model descriptions are valid. With this in mind, we record the concentration in both the stochastic and deterministic regions for each iteration of the algorithm and combine the concentrations together to give an average value for the concentration at each position. That is we take, for any point in the overlap region for any of the iterations of the algorithm, 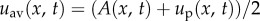, where *u*_av_ is the concentration in the overlap region, *u*_p_ is the concentration in the PDE-based region and *A* is the particle number in the compartment-based region. Otherwise, outside the regions covered by the overlap region, we use the deterministic and stochastic descriptions as usual. It is this combination of deterministic and stochastic descriptions that is plotted in figure 9.

Algorithm 2. **Algorithm for stochastic reaction–diffusion simulations with an adaptive interface using a compartment-based region and a PDE-based region.**

(i) Initialize and apply initial conditions. Set *t* := 0 and *k* := 0.
(ii) If *k* = *η*, where *η* is the checking interval, then check position of interface, otherwise proceed to step (iv).
(iii) If *A_i_* > *u*_max_ for 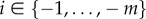, and *u_j_* > *u*_max_ for 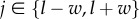, where the *l*th lattice point of the PDE lies on the interface *I*_1_, and *w* = *h*/Δ*x_p_* is the ratio of discretizations in *Ω*_c_ and *Ω*_p_, then update interface: 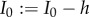.
  If 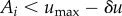 for 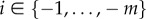 and 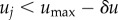 for 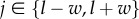, then update interface: 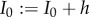.
  If *I*_0_ has been updated, then density in newly created region is equal to density of that region in previous description.
(iv) Implement one iteration of Algorithm 1. Increment *k* = *k* + 1. Return to step (ii) unless final time is reached.

[Boxed-text RSIF20160335BX2]Notable computational improvements are afforded by the hybrid model in comparison to the fully stochastic compartment-based model. Simulation time is decreased by a simulation-dependent factor of around 5. Note that the adaptive interface algorithm for the travelling wave simulations is significantly faster than the scenario with the fixed interface. Despite the cost associated with moving the interface, there is a large reduction in simulation time because the computationally cheaper PDE is solved on a larger part of the domain and the more computationally intensive stochastic model is restricted to a smaller region, in comparison to the hybrid model with a fixed interface (table 1).

**Table 1.** Computation times for each of the test problems, comparing the hybrid model with the fully stochastic model. Parameters used are as for figures 4, 6, 7, 9. Speed ups are given as a multiple of the fully stochastic time.

model	fully stochastic model (s)	hybrid model (s)	speed up
simple diffusion (IC: mass in [0,1])	1381.5	260.6	5.3×
morphogen gradient	2721.6	518.0	5.3×
travelling wave (fixed interface)	3133.3	688.1	4.6×
travelling wave (adaptive interface)	3133.3	527.6	5.9×

### 3.2. Sensitivity analysis

**Figure 10.**
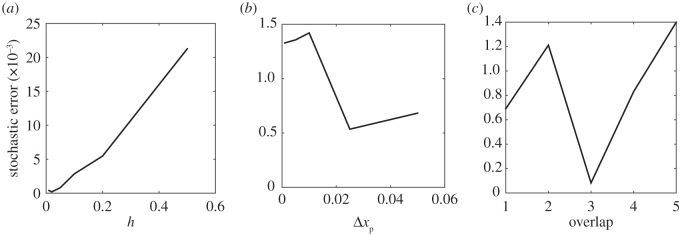
Sensitivity of the hybrid method to varying algorithm parameters: compartment size, *h*, PDE discretization, Δ*x*_p_, and number of compartments in the overlap region, *m*. The stochastic error shown here is the absolute value of the difference between several repeats of a stochastic simulation and the analytic solution. Parameters used for simulations were as for figure 3, with a total of 1000 particles and 10 repeats. Panels (*a*), (*b*) and (*c*) show the relative error for *h*, Δ*x*_p_ and *m*, respectively.

We demonstrate robustness of the coupling algorithm to choices of the algorithm parameters *h*, the compartment width, and Δ*x*_p_, the PDE discretization, showing how the total error varies as a function of these parameters. Because we are also able to vary the size of the overlap region in our coupling algorithm, we also demonstrate the effects of varying the number of compartments in this region. As the test problem here, we use simple diffusion with the same step-function initial condition as in figure 3 given by equation (3.2). The results are presented in figure 10. The total error *E* is calculated by summing the absolute value of the point-wise differences between the analytical and the hybrid solutions at the centre of each compartment in *Ω*_c_ and equivalently in *Ω*_p_. The error is shown for a single time point, at *t* = 1.

As *h* increases, the error value increases due to the smaller number of compartments used and the corresponding larger size of each compartment. However, this is the behaviour we would expect and is also seen in the fully stochastic model, as shown by the derivation in Appendix A. With varying Δ*x*_p_, the magnitude of the stochastic error remains approximately constant. Similarly, the error is independent of changes in the number of compartments in the overlap region.

### 3.3. Variance coupling

In previous sections, we have demonstrated that our hybrid coupling method correctly matches the mean behaviour of the compartment-based model and the deterministic PDE. In other similar coupling schemes, it has been of interest to ensure higher order moments match between the two modelling regimes. This has been successfully achieved with an overlap region [3].

**Figure 11.**
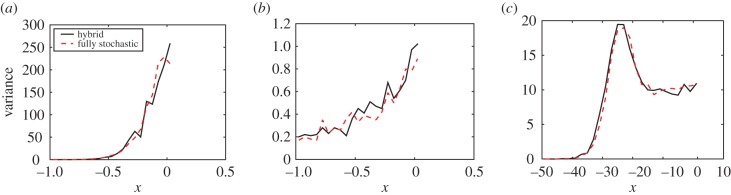
Variance of the particle density for each of the test problems in §2.1. Panels (*a*), (*b*) and (*c*) show the variance for diffusion (with initial condition as in (3.2)), morphogen gradient formation and the travelling wave (with fixed interface), respectively. The black full line shows the hybrid model and the dashed red line shows the fully stochastic model results. Parameters used for simulations were as previously: 100 repeats up to time *t* = 10 for panel (*a*), 100 repeats up to time *t* = 20 for panel (*b*) and 1000 repeats up to time *t* = 20 for panel (*c*). (Online version in colour.)

Our coupling methodology naturally employs an overlap region between the different modelling regimes. We found that the variance of our hybrid method agrees with that observed from simulations of the fully stochastic compartment-based model, as shown in figure 11.

## 4. Discussion

### 4.1. Summary

In this article, we have presented a novel hybrid algorithm for coupling a stochastic compartment-based model with a deterministic PDE model for reaction–diffusion systems. This technique is helpful for simulating reaction–diffusion systems, providing most benefit in comparison with existing methods in cases where a detailed description is necessary in a part of the domain of interest, but there are computational restrictions preventing the use of the detailed stochastic model throughout the domain. We utilize an overlap region where both modelling descriptions are valid. To perform the coupling, we apply a flux-based condition at one interface and a Dirichlet-type condition at the other interface. Furthermore, we justified mathematically the particular form of the boundary conditions used.

Biochemical systems where reaction–diffusion modelling approaches have been applied are found widely in the natural world from population ecology [21], to the spread of epidemics [21], to cell biology such as calcium signalling [2], and wound healing [27]. In particular, we focused on systems with multiple scales where detailed modelling is required in a certain region, but it might prove computationally wasteful to apply that method throughout the domain. Such systems occur frequently in a biological context due to the multiscale nature of biological systems [28].

The hybrid algorithm that we have developed was robustly tested and demonstrated by applying it to several biologically motivated problems in §3.1. There are noteworthy improvements in simulation time in comparison to a fully stochastic model, including a decrease in simulation time by approximately a factor of 5 when applied to a suite of standard test problems. The performance of this hybrid algorithm and the error compared to an analytic solution were analysed and explained.

At low particle numbers, a deterministic modelling method may no longer be appropriate and a stochastic method should be applied to account for the variation. There are disadvantages to the stochastic methods too; in particular they can require long simulation times. In order to make best use of the complementary advantages of deterministic and stochastic models, multiscale hybrid models are becoming increasingly widespread, particularly in applications relating to reaction–diffusion systems. We have presented our own hybrid coupling algorithm to segue between stochastic compartment-based models and deterministic PDE-based models. Further computational improvements have been reached by adding an adaptive interface to the algorithm.

## Appendix A. Linear dependence on compartment size

We demonstrate linear dependence of the error between the density of the compartment-based model and the mean-field PDE density, by performing a Taylor expansion in the compartment size *h*. From the master equation for the *j*th compartment, assuming only diffusion and no reactions, we have

where *d* is the jump rate between compartments. Applying a Taylor expansion in space

Taking a jump rate *d* = *D*/*h*^2^, we recover the diffusion equation with diffusion coefficient *D* and an error term . This gives an error between the compartment-based model and the mean-field diffusion equation linear in the compartment spacing *h*, as observed in figure 10.

## References

[1] Erban R, Flegg M, Papoian G 2014 Multiscale stochastic reaction–diffusion modeling: application to actin dynamics in filopodia. Bull. Math. Biol. 76, 799–818. (10.1007/s11538-013-9844-3) 23640574

[2] Rüdiger S, Shuai JW, Huisinga W, Nagaiah C, Warnecke G, Parker I, Falcke M 2007 Hybrid stochastic and deterministic simulations of calcium blips. Biophys. J. 93, 1847–1857. (10.1529/biophysj.106.099879) 17496042 PMC1959544

[3] Franz B, Flegg M, Chapman J, Erban R 2013 Multiscale reaction-diffusion algorithms: PDE-assisted Brownian dynamics. SIAM J. Appl. Math. 73, 1224–1247. (10.1137/120882469)

[4] Keller E, Segel L 1971 Traveling bands of chemotactic bacteria: a theoretical analysis. J. Theor. Biol. 30, 235–248. (10.1016/0022-5193(71)90051-8) 4926702

[5] Schlögl F 1972 Chemical reaction models for non-equilibrium phase transitions. Z. Phys. 253, 147–161. (10.1007/BF01379769)

[6] Franz B, Erban R 2013 Hybrid modelling of individual movement and collective behaviour. In Dispersal, individual movement and spatial ecology, pp. 129–157. Berlin, Germany: Springer.

[7] Flegg M, Chapman J, Erban R 2012 The two-regime method for optimizing stochastic reaction–diffusion simulations. J. R. Soc. Interface 9, 859–868. (10.1098/rsif.2011.0574) 22012973 PMC3306650

[8] Flekkøy E, Feder J, Wagner G 2001 Coupling particles and fields in a diffusive hybrid model. Phys. Rev. E 64, 066302 (10.1103/PhysRevE.64.066302) 11736271

[9] Moro E 2004 Hybrid method for simulating front propagation in reaction-diffusion systems. Phys. Rev. E 69, 060101 (10.1103/PhysRevE.69.060101) 15244531

[10] Ferm L, Hellander A, Lötstedt P 2010 An adaptive algorithm for simulation of stochastic reaction–diffusion processes. J. Comput. Phys. 229, 343–360. (10.1016/j.jcp.2009.09.030)

[11] Yates C, Flegg M 2015 The pseudo-compartment method for coupling partial differential equation and compartment-based models of diffusion. J. R. Soc. Interface 12, 20150141 (10.1098/rsif.2015.0141) 25904527 PMC4424691

[12] Spill F, Guerrero P, Alarcon T, Maini P, Byrne H 2015 Hybrid approaches for multiple-species stochastic reaction–diffusion models. J. Comput. Phys. 299, 429–445. (10.1016/j.jcp.2015.07.002) 26478601 PMC4554296

[13] Gardiner C 1985 Handbook of stochastic methods, vol. 3 Berlin, Germany: Springer.

[14] Donev A, Bell J, Garcia A, Alder B 2010 A hybrid particle-continuum method for hydrodynamics of complex fluids. Multiscale Model. Simul. 8, 871–911. (10.1137/090774501)

[15] Erban R, Chapman J 2007 Reactive boundary conditions for stochastic simulations of reaction–diffusion processes. Phys. Biol. 4, 16–28. (10.1088/1478-3975/4/1/003) 17406082

[16] Taylor PR, Baker RE, Yates CA 2015 Deriving appropriate boundary conditions, and accelerating position-jump simulations, of diffusion using non-local jumping. Phys. Biol. 12, 016006 (10.1088/1478-3975/12/1/016006) 25514045

[17] Gillespie DT 1977 Exact stochastic simulation of coupled chemical reactions. J. Phys. Chem. 81, 2340–2361. (10.1021/j100540a008)

[18] Fisher RA 1937 The wave of advance of advantageous genes. Ann. Eugenics 7, 355–369. (10.1111/j.1469-1809.1937.tb02153.x)

[19] Grenfell BT, Bjørnstad ON, Kappey J 2001 Travelling waves and spatial hierarchies in measles epidemics. Nature 414, 716–723. (10.1038/414716a) 11742391

[20] Keener J, Sneyd J 1998 Mathematical physiology, vol. 8 Berlin, Germany: Springer.

[21] Murray J 2002 Mathematical biology, vol. 1 Berlin, Germany: Springer.

[22] Robinson M, Flegg M, Erban R 2014 Adaptive two-regime method: application to front propagation. J. Chem. Phys. 140, 124109 (10.1063/1.4868652) 24697426

[23] Brunet E, Derrida B 1999 Microscopic models of traveling wave equations. Comput. Phys. Commun. 121–122, 376–381. (10.1016/S0010-4655(99)00358-6)

[24] Breuer H, Huber W, Petruccione F 1994 Fluctuation effects on wave propagation in a reaction-diffusion process. Phys. D Nonlinear Phenom. 73, 259–273. (10.1016/0167-2789(94)90161-9)

[25] Panja D 2004 Effects of fluctuations on propagating fronts. Phys. Rep. 393, 87–174. (10.1016/j.physrep.2003.12.001)

[26] Ho CP 2012 Multi-scale reaction diffusion simulations in biology. Master's thesis, University of Oxford, Oxford, UK.

[27] Sherratt J, Murray J 1990 Models of epidermal wound healing. Proc. R. Soc. Lond. B 241, 29–36. (10.1098/rspb.1990.0061) 1978332

[28] Meier-Schellersheim M, Fraser I, Klauschen F 2009 Multiscale modeling for biologists. Wiley Interdiscip. Rev. Syst. Biol. Med.. 1, 4–14. (10.1002/wsbm.33) 20448808 PMC2862646

